# Pharmacodynamic analysis of apremilast in Japanese patients with moderate to severe psoriasis: Results from a phase 2b randomized trial

**DOI:** 10.1111/1346-8138.15596

**Published:** 2020-09-10

**Authors:** Shinichi Imafuku, Osamu Nemoto, Yukari Okubo, Mayumi Komine, Peter Schafer, Rosemary Petric, Mamitaro Ohtsuki

**Affiliations:** ^1^ Fukuoka University Fukuoka Japan; ^2^ Kojinkai Sapporo Skin Clinic Sapporo Japan; ^3^ Tokyo Medical University Tokyo Japan; ^4^ Jichi Medical University Shimotsuke Japan; ^5^ Celgene Corporation Summit New Jersey USA

**Keywords:** apremilast, biomarker analysis, cytokines, pharmacodynamics, psoriasis

## Abstract

We evaluated the pharmacodynamic effects of apremilast in 69 patients who were included in biomarker subanalyses of a phase 2b study that demonstrated the long‐term safety and efficacy of apremilast in Japanese adults with moderate to severe psoriasis. The association between cytokine levels and Psoriasis Area and Severity Index (PASI) improvement was evaluated using linear regression and Spearman’s rank correlation coefficient analysis. At baseline, median plasma levels of interleukin (IL)‐17A, IL‐17F and IL‐22 were elevated versus reference values for healthy individuals, whereas tumor necrosis factor‐α levels were close to normal. With apremilast 30 mg b.i.d., there were significant associations between percentage change in PASI score and percentage change in IL‐17A, IL‐17F and IL‐22 levels at week 16. Findings demonstrate that the efficacy of apremilast in psoriasis is associated with inhibition of key cytokines involved in the pathology of psoriasis.

## INTRODUCTION

Apremilast is approved in Japan for the treatment of adult patients with plaque psoriasis with an inadequate response to topical therapies and adult patients with psoriatic arthritis. Through phosphodiesterase 4 inhibition, apremilast selectively inhibits the production of key cytokines that regulate inflammation.^1^, ^2^

Exploratory analyses from biomarker substudies of patients with moderate to severe psoriasis in the Efficacy and Safety Trial Evaluating the Effects of Apremilast in Psoriasis 2 (ESTEEM 2) study in North America and Europe, and in a phase 2b study in Japan, demonstrated reductions in key cytokines related to psoriasis pathology, namely 2 isoforms of interleukin (IL)‐17 (IL‐17A and IL‐17F), IL‐22 and tumor necrosis factor (TNF)‐α, with apremilast but not placebo.^3^ To further understand the relationship between the efficacy of apremilast and reductions in key cytokines, this exploratory biomarker analysis examined the correlation between clinical efficacy and plasma cytokine levels.

## METHODS

### Study design and patient population

This was a substudy of the phase 2b, multicenter, randomized, double‐blind, placebo‐controlled trial that demonstrated the efficacy and safety of apremilast in adult Japanese patients (aged ≥20 years) with moderate to severe plaque psoriasis, defined as Psoriasis Area and Severity Index (PASI) score of 12 or more and body surface area involvement of 10% or more, that was considered inappropriate for topical therapy (based on severity of disease and extent of affected area) or that had not been adequately controlled by 4 weeks or more of prior therapy with one or more topical medications (or per label; ClinicalTrials.gov: NCT01988103). The study design and patient population were previously described.^4^ Briefly, patients were randomized (1:1:1) to receive placebo, apremilast 20 mg b.i.d. or apremilast 30 mg b.i.d. for 16 weeks. Patients initially randomized to placebo were then re‐randomized (1:1) to receive apremilast 20 mg b.i.d. or apremilast 30 mg b.i.d. through week 68, after which they participated in a 4‐week observational follow‐up period. This study was conducted according to the general ethical principles outlined in the Declaration of Helsinki and adhered to Good Clinical Practice, as denoted in the International Conference on Harmonisation, as well as Japanese Good Clinical Practice. All patients provided written informed consent before participating in the study, and a total of 74 patients provided separate written informed consent to participate in the biomarker analysis.

### Pharmacodynamics assessments

Blood samples for biomarker analysis were obtained at baseline, at weeks 2, 4, 16, 24 and 52, and at the week 72 follow‐up visit. The concentrations of four plasma cytokines, IL‐17A, IL‐17F, IL‐22 and TNF‐α, were quantified by immunoassay using ultrasensitive single‐molecule counting (SMC™) Erenna^®^ technology (Singulex^®^ Clinical Laboratory, Alameda, CA, USA), a variation of traditional enzyme‐linked immunosorbent assay that improves signal‐to‐noise ratios to provide enhanced sensitivity and dynamic range.

### Statistical analysis

Analyses were performed in the biomarker subset of the modified intention‐to‐treat (mITT) population, defined as randomized patients who received one or more dose of study medication and had a baseline value and one or more on‐treatment, post‐baseline values for any biomarker. Summary statistics of observed values and change from baseline for each biomarker were provided for all assessed time points and at week 16 using the last observation carried forward (LOCF) approach. Between‐treatment comparisons versus placebo were performed at weeks 2, 4 and 16 using an ancova model with treatment group as a factor and baseline biomarker value as a covariate. A post‐hoc sensitivity analysis evaluated changes in cytokine levels among the study groups using a two‐sided Wilcoxon rank sum test; this non‐parametric test was performed because the distributions for changes from baseline in cytokine levels were skewed and included a few outliers.

The relationship between percentage change from baseline in PASI score and percentage change from baseline in plasma biomarker levels at week 16 was assessed within treatment groups using a linear regression ancova model with baseline plasma cytokine level and percentage change from baseline in plasma cytokine level at week 16 as covariates. The interaction of treatment and percentage change in plasma cytokine levels was also evaluated using an ancova model with the same covariates and including treatment as a factor, and an interaction term of treatment and percentage change from baseline cytokine level at week 16. Post‐hoc Spearman’s rank correlation coefficients and scatter plots were provided to further evaluate the association between cytokine levels and PASI score at baseline, and percentage change from baseline in cytokine levels and PASI score at week 16.

## RESULTS

### Patients

Of the 74 patients who provided written informed consent to participate in the biomarker analysis, 69 patients from 21 centers had baseline and post‐baseline assessments of cytokine levels and were included in the biomarker subset (placebo, *n* = 23; apremilast 20 mg b.i.d., *n* = 22; apremilast 30 mg b.i.d., *n* = 24). Patient demographics and baseline disease characteristics in the biomarker subset (Table S1) were generally similar to the 254 patients included in the full mITT population.^4^

### Changes in plasma cytokine levels

Median baseline plasma cytokine levels were similar in the placebo, apremilast 20 mg b.i.d. and apremilast 30 mg b.i.d. treatment groups (Table S2). IL‐17A, IL‐17F and IL‐22 plasma levels were elevated at baseline compared with healthy control subjects evaluated in studies that used the same Erenna immunoassay for cytokine analysis, whereas TNF‐α levels in our study were similar to those in healthy individuals (Table S2). Significant improvements versus placebo were observed for IL‐17A with apremilast 20 mg b.i.d. and apremilast 30 mg b.i.d. at weeks 2 and 4 (Fig. S1a); for IL‐17F with apremilast 20 mg b.i.d. and apremilast 30 mg b.i.d. at weeks 2, 4 and 16 (Fig. S1b); for IL‐22 with apremilast 20 mg b.i.d. at weeks 2 and 4 and with apremilast 30 mg b.i.d. at weeks 2, 4 (Fig. S1c) and 16; and for TNF‐α with apremilast 20 mg b.i.d. at weeks 4 and 16 (Fig. S1d).^3^ Sensitivity analyses using Wilcoxon rank sum tests were generally consistent with the ancova results.

Reductions in plasma cytokine levels were generally maintained with ongoing apremilast treatment and were observed after switching from placebo to open‐label apremilast treatment through week 52.^3^

### Correlation between PASI improvement and plasma cytokine levels

**Figure 1:**
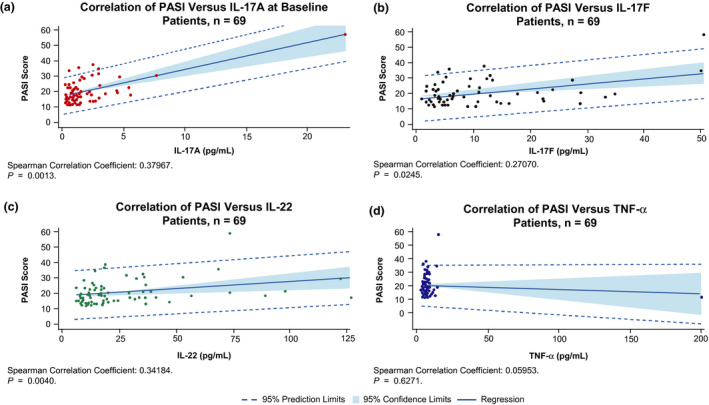
Correlation between Psoriasis Area and Severity Index (PASI) score and (a) interleukin (IL)‐17A, (b) IL‐17F, (c) IL‐22 and (d) tumor necrosis factor (TNF)‐α levels at baseline.

**Figure 2:**
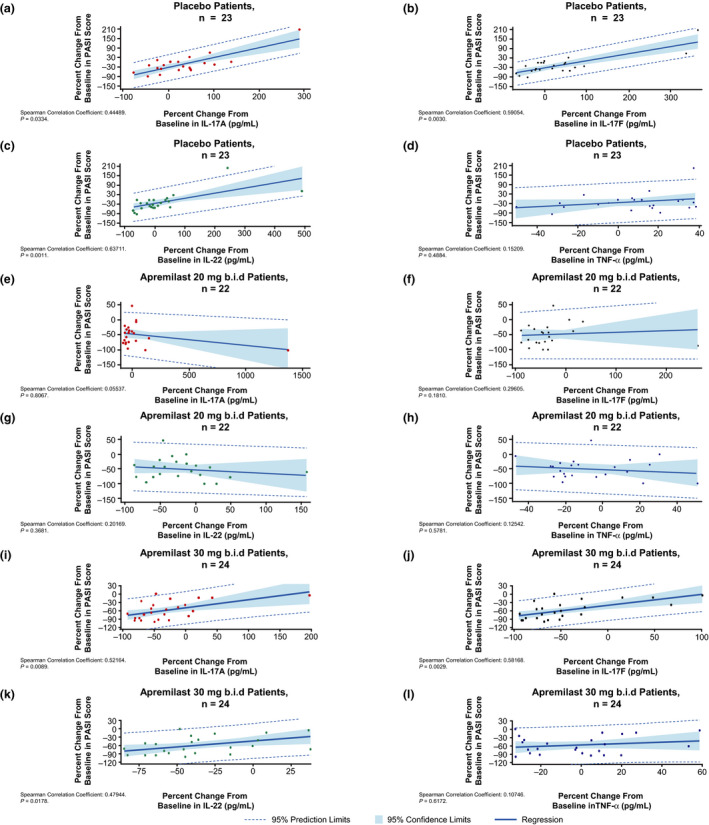
Correlations between percentage change from baseline at week 16 (last observation carried forward, LOCF) in Psoriasis Area and Severity Index (PASI) score with interleukin (IL)‐17A, IL‐17F, IL‐22 and tumor necrosis factor (TNF)‐α at week 16 in patients receiving (a–d) placebo b.i.d., (e–h) apremilast 20 mg b.i.d. and (i–l) apremilast 30 mg b.i.d.

Results from the ancova found significant and positive associations between percentage change from baseline at week 16 in PASI score and plasma levels of IL‐17A, IL‐17F and IL‐22 in patients who received placebo and in those treated with apremilast 30 mg b.i.d. (Table 1). Statistically significant interactions were observed for treatment and IL‐17A, IL‐17F and IL‐22, indicating that the relationships between PASI score and percent changes from baseline in the levels of these cytokines differed across the three treatment groups. There were no statistically significant associations between apremilast 20 mg b.i.d. and plasma levels of IL‐17A, IL‐17F and IL‐22. Associations between changes in TNF‐α, IL‐22 and PASI score at week 16 were not statistically significant across the three treatment groups. Sensitivity analyses using Spearman’s rank correlation coefficients confirmed the ancova findings, showing significant, moderate positive correlations between levels of IL‐17A, IL‐17F and IL‐22 and PASI score at baseline (Fig. 1), and significant positive associations between the percentage change from baseline in IL‐17A, IL‐17F and IL‐22 levels and percentage change from baseline in PASI score at week 16 in the placebo and apremilast 30 mg b.i.d. groups (Fig. 2).

**Table 1:** Association between PASI improvement and plasma cytokine levels at week 16^†^

Cytokine	Placebo, *n* = 23		Apremilast 20 mg b.i.d., *n* = 22		Apremilast 30 mg b.i.d., *n* = 24		Interaction
	Estimate (95% CI)	*P*	Estimate (95% CI)	*P*	Estimate (95% CI)	*P*	*P* ^‡^
IL‐17A	0.62 (0.39–0.84)	<0.0001	−0.04 (−0.09–0.02)	0.1782	0.33 (0.12–0.55)	0.0040	<0.0001
IL‐17F	0.46 (0.31–0.60)	<0.0001	0.07 (−0.17–0.31)	0.5486	0.48 (0.24–0.71)	0.0004	0.0063
IL‐22	0.34 (0.17–0.51)	0.0005	−0.17 (−0.52–0.18)	0.3208	0.54 (0.10–0.97)	0.0185	0.0264
TNF‐α	0.80 (−0.44–2.04)	0.1932	−0.20 (−0.95–0.54)	0.5721	0.22 (−0.38–0.81)	0.4536	0.2230

^†^ Estimated coefficient from univariate linear regression analysis of cytokine percent change from baseline calculated by ancova model with baseline cytokine level and cytokine percent change from baseline at week 16 (last observation carried forward, LOCF) as covariates.

^‡^ Interaction *P*‐value calculated using an ancova model with treatment as a factor, baseline cytokine level and cytokine percent change from baseline at week 16 (LOCF) as covariates, and interaction of treatment and cytokine percent change from baseline at week 16 (LOCF). CI, confidence interval; IL, interleukin; PASI, Psoriasis Area and Severity Index; TNF, tumor necrosis factor.

## DISCUSSION

Reductions in plasma cytokine levels observed with apremilast treatment in this study are consistent with prior analyses of patients enrolled in ESTEEM 2, in which apremilast treatment was associated with significant reductions from baseline in plasma levels of IL‐17A, IL‐17F, IL‐22 and TNF‐α at weeks 4 and 16 compared with placebo.^3^ The sustained cytokine reductions with apremilast treatment mirror the sustained efficacy of apremilast observed in apremilast clinical trials of patients with moderate to severe psoriasis.^4^, ^5^, ^6^

Significant and positive associations were observed between apremilast‐related improvements in PASI score and decreases in plasma levels of IL‐17A, IL‐17F and IL‐22. The correlation between changes in TNF‐α levels and PASI improvement at week 16 was not statistically significant. Patients in this analysis had TNF‐α levels at baseline that were similar to healthy individuals, and the magnitude of change from baseline in TNF‐α levels was subtle; thus, it is possible that reductions in plasma TNF‐α levels after apremilast treatment were too subtle to demonstrate an association with PASI improvement.

A prior analysis of predictive algorithms using data from ESTEEM 2 found that IL‐17 was the most important predictor of PASI response and identified synergistic effects of IL‐17, IL‐22 and TNF‐α on PASI response.^3^ Our analysis further establishes the relationships between reductions in plasma cytokine levels and PASI improvement with apremilast treatment. Taken together, both preclinical and pharmacodynamic findings demonstrate that apremilast has synergistic effects on key cytokines involved in the pathology of psoriasis through which apremilast provides its therapeutic effects.^7^, ^8^

## CONFLICT OF INTEREST

S. I. received research funds, consultancy fees and speaker fees from Celgene, Kyowa Hakko Kirin, LEO Pharma, Maruho and Novartis. Y. O. received grants from Eisai, Kyowa Hakko Kirin, Maruho and Mitsubishi‐Tanabe. P. S. and R. P. were employed by Bristol‐Myers Squibb, and Celgene at the time of study conduct. M. O. received grants and personal fees from AbbVie, Eisai, Mitsubishi‐Tanabe and Novartis. The remaining authors have nothing to disclose.

## Supporting information


**Figure S1.** Median percentage change from baseline in (a) interleukin (IL)‐17A, (b) IL‐17F, (c) IL‐22 and (d) tumor necrosis factor (TNF)‐α over 52 weeks. Patients initially randomized to placebo were re‐randomized to apremilast 20 mg b.i.d. or apremilast 30 mg b.i.d. at week 16. At weeks 2, 4 and 16, two‐sided *P*‐values were calculated based on ancova and Wilcoxon rank sum tests to investigate the percentage change difference between placebo and apremilast groups. *P < 0.05, **P < 0.01, ***P < 0.001 and ****P < 0.0001 versus placebo based on ancova. PASI, Psoriasis Area and Severity Index. Adapted from Garcet *et al*., with permission.

**Table S1.** Patient demographics and baseline disease characteristics in the biomarker subset
**Table S2.** Median plasma cytokine levels at baseline in the current study and serum or plasma cytokine levels in studies of healthy individuals
