# Supplementary material for: Pharmacodynamic analysis of apremilast in Japanese patients with moderate to severe psoriasis: Results from a phase 2b randomized trial
Source: J Dermatol. 2020 Sep 10;48(1):80–4. doi: 10.1111/1346-8138.15596 (PMC7821327; doi:10.1111/1346-8138.15596)
Supplement: Supplementary file 2 — Table S1. Patient demographics and baseline disease characteristics in the biomarker subset Table S2. Median plasma cytokine levels at baseline in the current study and serum or plasma cytokine levels in studies of healthy individuals [file JDE-48-80-s002.docx]

**Supplementary Appendix**

**Figure S1.** Median percentage change from baseline in (a) interleukin (IL)-17A, (b) IL-17F, (c) IL-22, and (d) tumor necrosis factor (TNF)-α over 52 weeks. Patients initially randomized to placebo were re-randomized to apremilast 20 mg b.i.d. or apremilast 30 mg b.i.d. at week 16. At weeks 2, 4, and 16, 2-sided *P* values were calculated based on ANCOVA and Wilcoxon rank sum tests to investigate the percentage change difference between placebo and apremilast groups. **P* < 0.05, ***P* < 0.01, ****P* < 0.001, and *****P* < 0.0001 versus placebo based on ANCOVA. PASI, Psoriasis Area and Severity Index. Adapted with permission from Garcet S, Nograles K, Correa da Rosa J, Schafer PH, Krueger JG. Synergistic cytokine effects as apremilast response predictors in patients with psoriasis. *J Allergy Clin Immunol.* 2018;142(3):1010-1013.e1016.

**Table S1.** Patient demographics and baseline disease characteristics in the biomarker subset

| **Characteristic** | **Placebo**  **n = 23** | **Apremilast  20 mg b.i.d.**  **n = 22** | **Apremilast  30 mg b.i.d.**  **n = 24** |
| --- | --- | --- | --- |
| Age, mean, years | 50.7 | 54.0 | 51.5 |
| Male, n (%) | 16 (69.6) | 15 (68.2) | 17 (70.8) |
| BMI, mean (SD), kg/m^2^ | 25.9 (5.0) | 25.6 (4.0) | 25.0 (3.8) |
| Weight, mean (SD), kg | 71.8 (12.7) | 70.3 (12.2) | 69.8 (12.7) |
| Duration of psoriasis, mean (SD), years | 13.6 (11.0) | 14.4 (11.7) | 17.0 (9.9) |
| PASI score (0–72), mean (SD) | 19.6 (7.6) | 21.6 (10.4) | 20.0 (5.7) |
| PASI score >20, n (%) | 6 (26.1) | 8 (36.4) | 12 (50.0) |
| BSA, mean (SD), % | 27.6 (11.9) | 30.8 (19.8) | 29.7 (11.1) |
| BSA >20%, n (%) | 15 (65.2) | 10 (45.5) | 19 (79.2) |
| sPGA=1 (minimal) | 1 (4.3) | 0 | 0 |
| sPGA=2 (mild), n (%) | 7 (30.4) | 5 (22.7) | 5 (20.8) |
| sPGA=3 (moderate), n (%) | 11 (47.8) | 12 (54.5) | 16 (66.7) |
| sPGA=4 (marked), n (%) | 4 (17.4) | 5 (22.7) | 3 (12.5) |
| sPGA=5 (severe), n (%) | 0 | 0 | 0 |
| Prior use of conventional systemic medications,  n (%) | 7 (30.4) | 11 (50.0) | 10 (41.7) |
| Prior use of biological therapies, n (%) | 3 (13.0) | 2 (9.1) | 0 |
| BMI, body mass index; BSA, psoriasis-involved body surface area; PASI, Psoriasis Area and Severity Index; SD, standard deviation; sPGA, Static Physician Global Assessment. | | | |

**Table S2.** Median plasma cytokine levels at baseline in the current study and serum or plasma cytokine levels in studies of healthy individuals

| **Cytokine** | **Patient With Moderate to Severe Psoriasis** | | | **Healthy Individuals** | | |
| --- | --- | --- | --- | --- | --- | --- |
|  | **Current Study** | | | **Todd  et al, *Cytokine*, 2013^§^** | **Schofield  et al, *Bioanalysis*, 2016^‡^** | **Shuckla et al, *Journal of Immunological Methods*, 2013^‡^** |
|  | **Placebo**  **n = 23** | **Apremilast 20 mg b.i.d.**  **n = 22** | **Apremilast 30 mg b.i.d.**  **n = 24** | **n = 32** | **n = 19** | **n = 10** |
| Median IL-17A, pg/mL | 1.4 | 1.1 | 1.2 | 0.2 | 0.3 | — |
| Median IL-17F, pg/mL | 8.3 | 5.3 | 6.4 | — | 0.7 | — |
| Median IL-22, pg/mL | 20.0 | 17.3 | 15.6 | — | — | 3.3^¶^ |
| Median TNF-α, pg/mL | 6.2 | 6.9 | 5.5 | 3.1 | — | — |
| **^§^**Cytokine levels were measured from plasma samples.  **^‡^**Cytokine levels were measured from serum samples.  ^¶^Represents mean concentration of IL-22.  IL-17A, interleukin 17A; IL-17F, interleukin 17F; IL-22, interleukin 22; TNF-α, tumor necrosis factor-α. | | | | | | |
